# Supplementary material for: Are carbon emissions trading and green financial instruments synergistic? -Comprehensive quantitative research based on content analysis
Source: PLoS One. 2024 Mar 7;19(3):e0298601. doi: 10.1371/journal.pone.0298601 (PMC10919601; doi:10.1371/journal.pone.0298601)
Supplement: S1 Data — (ZIP) [file pone.0298601.s001.zip › Data/地级市Z_F工作报告.zip!/╣π╢1⁄2/╒┐╜¡╩╨/└┤╫╘╣1⁄2╓┌║┼í╛┬φ┐╦╩2╛▌═°í┐_2004.docx]

各位代表：

　　我代表市人民政府向大会作政府工作报告，请予审议，并请市政协委员和其他列席会议的同志提出意见。

　　2003年工作回顾

　　2003年是湛江经济和社会发展不平凡的一年。一年来，在胡锦涛总书记视察湛江巨大政治动力激励下，在中共湛江市委的正确领导下，在市人大及其常委会和市政协的监督支持下，市政府以邓小平理论和“三个代表”重要思想为指导，贯彻落实党的十六大、十六届三中全会精神，积极实施“工业立市、以港兴市”发展战略，紧紧依靠全市人民，一手抓防治非典不放松，一手抓经济建设不动摇，按照建设现代化新兴港口工业城市的目标要求，全力推动经济和社会协调发展，主要经济指标增幅均创“九五”以来最好水平，精神文明建设、民主法制建设和社会各项事业取得新成绩，实现了市十一届人大一次会议确定的全年目标任务。

　　综合经济实力跃上新台阶。全市生产总值520.97亿元，增长10.1%，是1996年以来生产总值首次实现两位数增长。其中，第一产业增加值110.99亿元，增长3.4%；第二产业增加值221.97亿元，增长15.2%；第三产业增加值188.01亿元，增长8.5%。三次产业比例由上年的23.139.837.1变为21.342.636.1，经济结构有所优化。全市人均生产总值突破1000美元关口，跨上一个重要台阶。

　　“工业立市”初见成效。工业主导地位增强。第二产业在三次产业中增幅最高、增速最快，工业总产值535.92亿元，增长17.9%；工业增加值192.89亿元，增长16.5%，其中规模以上工业增加值146.97亿元，增长17.7%。工业增长对生产总值增长的贡献率为58%，拉动生产总值增长5.9个百分点。规模以上工业经济效益综合指数达175.1，提高10.9点。全社会用电量34.25亿千瓦时，增长16.1%。国企改革不断深化。国有企业改制面达到90%，部分企业完成了产权多元化改造；“冠豪高新”成功上市。地方15家糖厂全面进行产权制度改革，多年拖欠农民甘蔗款问题正妥善解决，糖业开始恢复生机；市属40家企业公开挂牌，实行产权转让；11家药厂引入民资或外资实施GMP改造；国有劣势企业加快退出市场，17家企业完成破产立案或破产前期工作，关闭了16家“三无”企业。国有粮食企业改革启动。县域工业发展加快。认真做好工业发展规划，积极引进项目。各县(市、区)共引入工业项目115个，总投资24.28亿元，其中37个建成投产；完成工业总产值249.4亿元，增长20.1%。非公经济发展迅速。全市新注册登记个体工商户1.5万户，增长11.4%，注册资本累计7.8亿元，增长27.3%；新发展私营企业1099户，增长32.7%，投入资本累计23.1亿元，增长58.8%；民营经济增加值278.19亿元，增长12.5%，占全市生产总值的53.4%，非公经济成为国民经济的重要主体。实施名牌战略取得成效。获得国家免检产品3个，省名牌产品17个、著名商标5个。

　　农村经济稳步发展。农业总产值180.4亿元，增长3.7%。农业布局和结构逐步优化。调减糖蔗种植面积11.05万亩，增加优质水果、外运菜、蚕桑、花卉等高效作物种植面积12.35万亩。农业产业化稳步推进。29家市级以上农业龙头企业销售收入47.46亿元，创汇5806万美元，带动农户17.5万户，户均增收2894元。新建和扩建水产品加工企业17家，新增加工能力8万吨；水产品加工量14.2万吨，产值23.6亿元，分别增长26.4%和28.2%。农业生产条件不断改善。续建和新建江洲围海堤、南渡河船闸、大水桥水库等一批水利工程；解决农村60.8万人饮水难工程进展顺利；改造中低产田8600亩；完成镇通村委会公路硬底化突破1000公里；基本完成9个村委会“大禹杯”项目和70个“千村扶贫工程”项目建设；5万吨国家储备粮湛江库项目建成使用。农业标准化取得新成效。已建立9个国家级或省级农业标准化示范区；农产品有12个通过国家绿色食品认证，23个被评为国家名优产品，4个被授予“广东省名牌产品”称号；26家水产品进出口企业获得欧盟、美国、韩国等国际认证；农产品出口总额2.69亿美元，增长98%，其中水产品出口创汇2.23亿美元，增长1倍多。海洋水产业发展较快。海水养殖产量34.35万吨，产值24.9亿元，分别增长6%和14%。对虾已形成育苗、养殖、销售、加工、出口一体化经营的产业链。由民营企业恒兴集团承担的国家863计划海水养殖种子工程南方基地建成投产，提高了海水养殖科技水平。畜牧业发展势头良好。完成产值34.07亿元，增长4.1%；形成了“广海鸡"、“嘉仙鸡”等多个品牌；饲料加工业产值25亿元，增长14%。林业发展步伐加快。完成造林27.88万亩，林业产值4.72亿元，增长7.8%。农村税费改革成效明显。清理整顿和规范各项涉农收费，积极推进“并镇、并村、并校、减人”等配套改革，农民负担减负率达89%，极大地调动了广大农民生产经营的积极性。

　　重点项目建设顺利推进。全社会固定资产投资114.13亿元，增长19.5%。投资亿元以上的重点项目25个，全年累计完成投资30.8亿元。已有30万吨级油码头投入运行，华农和富虹大豆加工、华丽金DVD—R二期等5个项目建成投产，新增产值7.3亿元；省道平杨线、遂六线和廉石线建成通车，全部实现市到县(市)通一级公路；东兴炼油扩建、奥里油发电厂、奥里油库、大中纺织凤梨纤维、冠豪特种涂布纸、海湾大桥、渝湛高速公路湛江段、疏港公路、30万吨级航道、20万吨级铁矿石码头、东海岛液化气冷冻储库等15个在建项目工程进展顺利。

　　开放型经济持续增长。外贸出口、吸收外商直接投资分别获得省二等奖和三等奖。外贸进出口总值19.35亿美元，增长62%。其中出口9.01亿美元，增长65%。国联、国溢等水产品加工企业在全国率先创建电子网络实时监管系统“2211”模式。各县(市、区)外贸出口均超过1400万美元，最高的达到2.7亿美元。利用外资项目50个，增长11.1%；实际吸收外商直接投资1.8亿美元，增长63.3%。有7个县(市、区)实际吸收外商直接投资超过500万美元，最高的达到2011万美元。举办’2003湛江工业博览会和到外地多次举办投资环境推介会、招商洽谈会，多方面招商引资，拓展国内外市场，提高了湛江知名度。

　　城市环境进一步优化。城市品位显著提升，获得“中国优秀旅游城市”称号。基本完成第五次《城市总体规划》修编和第一次《市域城镇体系规划》、《城市绿地系统规划》等编制工作。积极实施城市建设用地储备，共储备土地约5000亩，保障了重点项目用地。市政建设投入5.8亿元，实施项目30多个。寸金纪念广场、南桥公园、海田公园、霞山绿苑、湖光路口小游园、湖光路、寸金路、人民大道绿化及路灯、城区20万吨引水以及公路枢纽站场海田客运站等新建(改造)工程完成并投入使用；椹川大道、机场路、东新路、渔港公园和金沙湾观海长廊二期等新建(改造)工程进展顺利；赤坎污水处理厂主体工程完成。拆除违章和市政建设需拆除的建筑物55万平方米，新增绿地52万平方米，建成区绿化覆盖率上升到34.65%，绿地率达31.8%，人均公园绿地面积达到6.93平方米。三岭山森林公园被批准为省级森林公园；湖光岩风景区被评为国家4A级旅游区和国家级重点风景名胜区。城市环境卫生管理力度加大，市容市貌明显改观。开展“环保执法年”活动成效明显，环境质量保持位居全国前列，市区环境空气质量保持优良等级，城市生活垃圾无害化处理率达83.5%。

　　第三产业发展加快。努力采取措施，把非典对第三产业造成的影响降到最低。新的消费热点加快形成，数字家电、通讯、电子、汽车、住房等商品消费有较大增长。成功举办全国醒狮艺术邀请赛、酒吧街欢乐节、海鲜美食节，国贸B座、鑫海名城等大型商场开业和假日消费旺盛，带动消费平稳增长。社会消费品零售总额226.44亿元，增长10.1%。湛江港货物吞吐量3985万吨，增长11.1%。铁路货物发送量1174万吨，增长19.1%。邮电业务收入19.8亿元，增长8.6%。商品房销售面积44.09万平方米，增长50.2%。旅游业全年接待游客439万人次，总收入28.04亿元。

　　财政金融形势平稳。虽然因农村税费改革和扶持抗非典减免税费等政策因素造成部分税收减少，地方财政一般预算收入仍达到16.82亿元，增长10.5%，财政总收入43.54亿元，增长11.6%；地方财政一般预算支出44.1亿元，增长14.3%，实现财政收支平衡。年末金融机构存款余额540.05亿元，增长15.2%；贷款余额297.53亿元，增长5.8%。

　　人民生活水平有所提高。市区居民家庭人均可支配收入8235元，增长7.7%;在岗职工人均年工资11918元，增长13.3%；农民人均纯收入3687元，增长3.8%。城乡居民社会保障水平进一步提高,全市参加养老保险41.57万人，参加医疗保险34.74万人，参加失业保险33.12万人，分别完成省核定应参保标准的101%、104.5%和96.3%；征收社保费7.89亿元，其中企业养老和失业保险基金6.37亿元。低保面进一步扩大，已有9.8万困难群众纳入最低生活保障线范畴，低保覆盖面达1.4%。下岗失业人员再就业5.3万人，再就业率达77%，城镇新增就业岗位4.6万个，组织农村富余劳动力输出15.58万人。部分企业军转干部生活困难问题得到初步解决。

　　社会各项事业协调发展。教育：初步建立“以县为主”的义务教育管理体制和教师工资保障机制；巩固“普九”和扶贫助学工作取得较好效果，初中在校生增加13230人；中小学校布局调整顺利推进，撤并学校（教学点）146所，新建、扩建学校109所，其中已建成31所，改造老区学校100所，市区8所小学改造工程已部分完工；普通高中招生增长13.5％，在校生增长17.9%；示范性高中建设步伐加快；高考录取人数在全省排列第三位；高等学校办学规模不断扩大；中等职业教育强化了市场导向，培养了大批实用人才。科技：启动实施省级以上“火炬”和“星火”计划项目78项；新增省级以上高新技术企业14家，高新技术产品产值增长34％；以企业为主体的技术创新体系逐步完善；建立省级农业科技园；民营科技企业增加72家；市级科技计划项目全面引入市场机制实行公开招标；开展“振兴湛江——市民创意奖”活动。科技进步对工业增长的贡献率提高到44.5%。文化：编制《建设文化大市规划纲要》，启动建设文化大市工作；文化设施建设得到加强，建成粤西规模最大的湛江图书城，广播电视“村村通”工程进展顺利；“开心广场”等群众文化活动开展活跃，遂溪县被评为“中国醒狮之乡”；公布了第五批市级文物保护单位。医疗卫生：把保护人民身体健康和生命安全放在首位，全民动员抗击非典工作取得重大胜利；加强公共卫生体系建设，建立健全疫情信息网络和“120”急救指挥系统，市卫生监督、疾控中心完成体制改革；开展农村合作医疗试点工作，参加农村合作医疗22.06万人；药品集中招标采购工作规范进行；农村住户卫生厕普及率45%，创建卫生村工作得到省的肯定。体育：群众性体育活动蓬勃开展，在市区主要公共场所增设大批健身设施；竞技体育成绩显著，参加省级以上比赛获得奖牌197枚，其中金牌78枚；成功承办省第六届大学生运动会；体育工作荣获省“突出贡献奖”。人口与计划生育：认真执行《人口与计划生育法》和《广东省人口与计划生育条例》，计生工作重心下移，抓好基层服务，完成了上级下达的人口控制指标，人口自然增长率控制在8.23‰以内，计划生育率为84.8%，出生率为13.26‰。殡葬改革：市新殡仪馆建成使用，全年火化率99.1%。此外，人事制度改革深化，公务员考录制度逐步规范，市直全额拨款事业单位补充人员实行公开考试聘用。统计信息网络建成，统计、城调部门为经济社会发展提供了大量有价值的信息资料。审计部门加强了对财政资金、资产的监督。物价部门坚持把好物价关。第一部《湛江市志》完稿即将出版。档案依法规范化管理进一步加强。侨务、外事、对台等部门扩大与港澳台和国外的经济文化交流。气象、地震、减灾防灾、民族、宗教、残疾人事业等各项工作取得新的成绩。

　　精神文明、民主法制和廉政建设深入推进。扎实有效地推进群众性精神文明创建活动，获得“广东省文明城市”称号。以“四通五改六进村”为主要内容的生态文明村建设取得新成效，评定全国文明村2条和省文明示范村3条、文明村15条。双拥共建与优抚安置、国防动员、人民防空、兵员征集和民兵预备役工作不断加强，军政军民更加团结，再次荣获“全国双拥模范城”称号。民主法制建设继续推进,各级政府依法行政、接受监督的规范性不断增强，自觉性不断提高。认真处理人民群众来信来访，积极办理人大代表议案、建议和政协委员提案，办结议案、建议257件和提案143件。大力推进社区建设，基层司法组织、普法教育、法律服务工作得到加强。整顿市场经济秩序和信用建设初见成效，规范有序、公平公正、健康安全的市场环境逐步形成。安全生产坚持常抓不懈，初步建立企业安全生产长效机制，促进安全生产形势进一步好转，没有发生重大安全事故。深入开展严打整治斗争，集中整治治安重点地区和突出问题，严厉打击各类违法犯罪活动，大力扫除“黄赌毒”等社会丑恶现象，扎实开展“禁毒03”工程，打击走私工作继续保持高压态势，坚持同“法轮功”邪教组织作斗争。开展交通客运市场专项整治，严厉打击扰乱运输市场秩序的不法分子。人民调解组织和工作制度逐步完善，积极预防并妥善处置群体性事件，深入排查防范和依法调处一批山林土地滩涂纠纷，各种社会矛盾逐步化解，社会治安形势保持稳定。软环境有较大改善，市政府行政服务中心实行市长、局长办公日和重点项目现场办公制度，行政效率有较大提高。工商、国税、地税部门积极发挥职能作用，扶持企业加快发展。海关、出入境检验检疫、海事、边检等口岸部门努力完善“大通关”机制，为外经贸发展发挥积极的推动作用。政府廉政建设责任制进一步落实，出台一系列强化管理、监督的制度，规范对行使权力的约束，严肃查处了一批违法违纪案件。

　　各位代表!过去一年的成就来之不易。这些成就是中共湛江市委正确领导的结果，是各级人民代表大会及其常务委员会和人民政协监督支持的结果，是中央、省驻湛单位和社会各方面关心支持的结果，是全市人民同心同德、艰苦奋斗的结果。在此，我代表市人民政府，向各级人大代表和政协委员，向在各个工作岗位上辛勤劳动、作出贡献的广大工人、农民、知识分子、干部、驻湛人民解放军、武警部队官兵、公安干警和社会人士，向所有关心和帮助湛江建设发展的港澳台同胞、海外侨胞和国际友人，向信任和支持政府工作的全市人民致以崇高敬意和诚挚感谢!

　　在充分肯定成绩、共同增强信心的同时，我们清醒地看到，我市经济社会发展与时代的进步、与省委省政府的要求、与全省经济社会发展的平均水平、与人民群众的愿望还有较大差距，还存在不少困难和问题，政府工作还有不少缺点。主要是：总体经济规模偏小，仅排名在全省第九位，生产总值增长速度比全省低3.5个百分点，人均生产总值只达到省平均水平的一半；工业化程度较低，第二产业占生产总值比重与全省平均相比低9.8个百分点；县域经济发展滞后，农村经济增速缓慢，农民增收难度加大；城镇化水平与全省平均相比低15个百分点；城市建设和管理中机制创新不足，项目进度不够快，市区交通秩序较乱，污水、垃圾处理落后；社会就业压力增大，社会保障能力较弱，城乡部分群众生产生活困难；政府部门办事效率和服务意识尚未适应加快发展的形势等。因此，我们必须时时保持忧患与责任意识，保持谦虚谨慎的作风，保持苦干实干的工作劲头，高度重视并认真解决上述问题，迎接新的挑战，开创新的局面，决不辜负人民的期望。

　　2004年工作安排

　　2004年市政府工作的指导思想是:以邓小平理论和“三个代表”重要思想为指导，全面贯彻党的十六大、十六届三中全会精神，坚持以人为本和全面、协调、可持续的科学发展观，认真落实市委八届二次全会的工作部署，以建设现代化新兴港口工业城市为总目标总任务，深入实施“工业立市、以港兴市”发展战略，加速工业化进程，高度重视“三农”问题，壮大县域经济总量，加强城市基础设施和生态环境建设。完善市场经济体制，努力扩大开放，优化经济结构，增强综合竞争力。关心人民群众切身利益，提高城乡居民生活水平和质量。全力振兴经济，发展社会事业，努力赶上全省率先基本实现社会主义现代化步伐。

　　今年经济社会发展的主要预期目标是：全市生产总值增长10%，其中第一产业增长3.5%，第二产业增长13.7%(其中工业增长14%)，第三产业增长9.5%；全社会固定资产投资增长15%；社会消费品零售总额增长10%；外贸出口增长10%；实际吸收外商直接投资增长15%；地方财政一般预算收入增长10%；居民消费价格指数上升2%左右；城镇登记失业率控制在3.5%以内；人口自然增长率控制在9.2‰以下；城镇居民人均可支配收入增长8%，农村居民人均纯收入增长4%。

　　为实现上述目标，必须做好以下工作：

　　一、继续强化工业主导地位，全力推进工业化进程

　　全力加快重点项目建设。今年安排重点项目28个，年度投资45亿元。完善重点项目管理责任制，年内确保包装材料尼龙薄膜、大中纺织凤梨纤维、冠豪特种涂布纸、喜利得八厂扩建、中油奥里油库等项目建成投产。积极支持东兴炼油扩建、奥里油发电厂等在建项目加快建设进度，实现预期目标。扎实做好湛江纸浆厂、华能东海岛电厂、韶钢沿海（湛江）钢铁基地、石油储备基地、输西南及珠三角成品油管道工程和宝满港区集装箱以及散装化肥专业化码头等重大项目前期工作。抓好重大项目储备，做到投产一批，建设一批，储备一批，增强发展后劲。跟踪落实’2003湛江工业博览会签订的生产性投资项目，力促这批项目早动工、早投产、早出效益。

　　着力培育临港石化和农海产品加工产业。以深水大港为依托，大力发展临港工业，努力争取国内外大型企业投资建立或增资扩大生产基地，发展相关配套产业，形成石油化工产业聚集带；积极支持东兴石油、中海油湛江公司两个全省工业50强企业发展壮大，增强带动作用，为现有石化企业加快发展创造条件；积极引进辐射力大、带动性强的大项目。运用市场机制对农海产品加工业进行优化整合，重点培育一批实力强、规模大、技术水平高的企业，使之做强做大，成为产业的龙头；大力抓好专业加工基地建设，完善配套专业市场、信息技术等服务业。力争用3年时间，使临港石化和农海产品加工两大产业对全市工业增长的拉动，从目前的9个百分点提高到20个百分点，成为我市工业化的领航产业。努力发展小家电和服装产业，通过整合品牌、专利等资源，形成区域性生产基地。

　　推进国家级开发区和省级试验区建设。支持湛江经济技术开发区调整功能，论证、申报延伸区，发展高新技术产业，发挥经济发展的排头兵作用。加快东海岛等5个省级经济开发试验区建设。市财政继续安排专项资金扶持，采取多种优惠政策和措施，降低进入企业的运营成本。要突出重点，形成特色，引进投资主体尤其是引进民营投资主体开发建设，做到开发一片，建成一片，不断滚动发展，办出效益，办出效应。加强基础设施和投资环境建设，使开发区、试验区成为集聚企业、项目和生产要素的工业基地，年内要力争一批项目建成投产，使之成为新的经济增长点。

　　支持重点企业做大做强。支持拥有自主知识产权、具有产业优势、农海产品精深加工和大中型等几类企业加大技改投入；抓好20项重点技改项目，争取其中10项列入省重点项目计划。积极扶持年销售收入超亿元企业发展壮大。促进银企合作，为重点企业在资本市场筹资融资做好服务，推动湛江港股份公司等优势企业上市。继续实施名牌带动战略，推动一批企业争创国家级、省级名牌；根据产品规模和效益，继续对获得国际认证和国家免检产品、国家和省名牌产品、驰名和著名商标的企业进行奖励。对重点工业企业实行动态管理，公布和表彰全市工业50强。

　　大力发展民营企业。出台《关于扶持民营经济发展的实施办法》，放宽市场准入，加强服务引导，落实政策措施，为民营企业发展创造良好的社会环境。积极引进有实力的民营企业来我市投资创业。着力扶持科技型、外向型和吸纳下岗人员再就业的民营企业加快发展。鼓励和支持发展农海产品加工、小家电、羽绒、工艺家具、花卉苗木等特色民营企业。打破区域、行业封锁和体制障碍，鼓励民营资本进入市政公用事业领域。用好省市中小企业技术改造、技术创新专项扶持资金，提高民营企业的技术研发能力。建立和完善信用担保及服务体系，拓宽融资渠道，解决民营企业融资难问题。建立民营企业投诉中心，对重点民营企业实行挂牌保护和收费监测报告制度，切实维护民营企业的合法权益。

　　二、统筹城乡发展，切实解决“三农”问题

　　努力促进农民增加收入。认真贯彻落实中共中央、国务院《关于促进农民增加收入若干政策的意见》，按照统筹城乡经济社会发展的要求，坚持“多予、少取、放活”的方针，高度重视农业基础地位，深化农村改革，调整农业结构，增加农业投入，强化对农业的支持保护，把增加农民收入放在事关全局的突出位置。既要激发广大农民群众艰苦创业的积极性，通过辛勤劳动走上富裕之路，又要动员各行各业树立大局意识，为农民增收贡献力量。切实巩固农村税费改革成果，精简镇、村机构和财政供养人员，严肃查处各种违规收费行为，防止农民负担反弹。按国务院要求，逐步降低农业税税率，平均每年降低1个百分点以上，五年内取消农业税。

　　壮大县域工业总量。工业是发展和壮大县域经济的龙头，是推进农村城镇化和农业产业化的核心。各地要做好规划，抓住重点，调整结构，积极发展资源型加工业和特色工业。引导民营企业和新项目集中建设，推动工业企业聚集。县（市、区）要加大招商引资力度，承接发达地区和市区的产业转移，每年新上一批工业项目，尽快壮大工业总量。镇级政府要根据当地资源状况，积极引入民资、外资发展加工型项目。

　　推进农业结构调整。以市场为导向，积极调整农业结构，提高农产品的竞争力和农业综合效益。落实粮食工作政府负责制，严格实行耕地保护制度，切实保护基本农田，确保全市粮食总量稳定。调整种养业结构，大力发展高产、优质、高效、生态、安全的农业，重点抓好外运菜、优质水果、南药、湛江鸡、雷州牛等30多个优势农产品，集聚资源、技术力量和项目资金，培育一批优势区域、基地和品牌，形成优势农产品产业带。发挥国家863计划海水养殖种子工程南方基地和省级良种场的示范带动作用，发展“名、优、特”海水养殖业和海洋制药业。加快建设硇洲、乌石、龙头沙等重点现代渔港经济区。加大力度做好北部湾渔民转产转业工作。推广种草养畜，积极发展奶牛业和优质畜禽，建设优质畜禽产品产业区，把畜牧业培育成为支柱产业。大力发展林业，年内造林22万亩，重点抓好沿海防护林优良无性系示范、红树林人工造林、纸浆原料林和种苗基地建设，继续在市区海滩种植红树林；发展花卉、苗圃产业，把麻章至遂溪公路两旁建成上规模、高档次的花卉基地和集散地；研究开发香根草。发展壮大高效出口创汇农业，推动农业增长方式转变。

　　加快农业产业化进程。大力推广“龙头企业＋农民合作经济组织＋基地＋农户”的发展模式，创新龙头企业与农民的联结机制。延伸速生林、糖蔗、对虾、珍珠、果菜、畜牧六大优势农业产业链条，完善产业化体系。建成一批规模较大、辐射面广、带动力强的特色农业示范基地。重点支持50个市级以上农业龙头企业做大做强。扶持发展农村合作经济组织，提高农业组织化程度。发挥供销社带动农民进入市场和湛江农垦的基地示范作用。推进农业标准化生产，扩大农业标准化示范区域，启动市级农业标准示范项目建设，年内建成农产品、水产品、畜产品和饲料等质量安全检验检测中心；积极实施“无公害食品行动计划”，推进市级蔬菜、水果、花卉、畜牧、水产等无公害农产品生产基地建设，组织申报国家绿色食品和省级以上名牌产品，研究建立“湛江特产”产品标识体系并完善认证与管理机制。扶持发展农海产品加工和流通企业，开发更多特色产品，开拓国内外市场，扩大经营规模，鼓励建立湛江名优农海产品专卖店。健全农业社会化服务体系和农产品市场体系，完善农业综合信息网络。加快农业机械化发展步伐。

　　加快农村城镇化步伐。促进形成政府引导、科学规划、市场运作、产业支撑的城镇化机制。加大城镇交通、电力、通讯、环境等基础设施建设力度，把发展工业、第三产业与发展小城镇结合起来，健全多元化投资体制，加强县(市)城区和全市19个中心镇规划建设，增强吸纳农村人口、带动农村发展的能力。推进户籍制度改革，逐步统一城乡户籍管理制度。发展城乡劳动力市场，加强对农民的职业技能和转移就业培训，促进城乡劳动者平等就业，加快农村富余劳动力有序转移。大力发展农村个体私营经济，并在工商、税收、金融、资源、人才等方面给予支持。

　　加强农业基础设施建设。集中财力，增加投入，突出重点，提高支农资金的使用效益。组织实施农业综合开发、中低产田改造和“大禹杯”项目。继续抓好雷州半岛西南部治旱和农村饮水解困工程。启动实施城乡水利防灾减灾工程建设，重点抓好市区45公里堤防工程。认真落实省、市人大代表提出的议案，抓好小型水库除险加固，继续解决好水库移民遗留问题；加快雷州半岛气象监测预警发布中心和人工影响天气基地建设。建设镇通村委会公路硬底化700公里。继续实施农村电网改造，力争省对三个县级市电力体制实行直供直管，把农电到户电价降到每千瓦时0.7元以下。

　　三、深化体制改革，增强发展活力

　　按照市委八届二次全会通过的《体制改革要点》的要求，统筹兼顾，协调推进，努力消除经济社会发展中的体制性障碍，重点在五个方面实现突破：

　　完善国有资产监管体制。组建市级国有资产管理机构，建立健全国有资产监督管理体系，理顺产权关系，推进政企分开、所有权和经营权分离，建立绩效考核奖惩机制，提高国有资产运营效率，实现保值增值。实施《国有企业领导人员经营决策行为十条禁令》，强化对国有企业经营者的监管。市属企业退休人员实行社会化管理服务。

　　建立现代产权制度。按照有进有退、扶优扶强、保值增值、强化监管的要求，加快以产权制度为核心的国有企业改革。推进投资主体多元化，发展混合所有制经济。湛江港完成港口体制改革，实行政企分开，组建湛江港务管理局和湛江港集团有限公司。年内完成所有地方国有糖厂产权制度改革；推进三星公司资产重组；加快半球集团品牌资产整合，引入民资建立新的经营载体；完成国有粮食企业的改制和重组；推进供销合作社的产权制度改革和开放办社。采取民事破产、依法关闭等手段，加快劣势企业退出市场。全面取消企业行政级别和原管理体制，深化企业人事制度改革。推行国有企业负责人业绩考核、离任审计；全面实行资产经营责任制和逐步推行企业经营者年薪制。

　　着力提高行政效率。各级政府要突出为市场主体服务和营造发展环境，强化社会公共管理职能。政府决策要充分采纳民意，提高科学化与民主化程度。大幅度减少行政审批事项，推进行政审批法制化和规范化。简化办事程序，缩短办事时间，提高行政效率。推进市县政府机构改革和机关后勤体制改革。

　　创新城市经营管理。积极实施城市建设用地储备和供应制度，政府垄断土地一级市场和按建设需要以公开拍卖方式定量定向供应土地。充分运用市场机制筹集城市建设资金，对户外广告、加油站、城区交通等特许经营权由行政审批改为全面公开拍卖。推进市政公用事业和政府投资工程管理模式的改革，实行政事分开、政企分开、建管分开，使城市公用企业成为市场主体。开放市政公用事业建设和运营市场，在为社会公众服务的公共交通、园林绿化、环卫、供水、排污、城市燃气等行业引入竞争机制，实行市场化运作。放宽城市公用事业准入条件，鼓励民资、外资参与建设和运营。推进市政公用产品价格改革，建立既有利于激励社会投资，又兼顾到市民承受能力的价格机制。

　　建立公共财政体制。把财政等公共资源更多地向社会管理和公共服务倾斜，使更多的民众得到公共利益。切实依法加强税收征管。全面实行收支两条线和部门预算、财政集中收付制度，规范部门收支行为。严肃财经纪律，规范收费行为，努力做到生财有道、聚财有方、用财有规，积极开展财政支出绩效评价工作。实行市直机关公务员工资、岗位津贴统发。全面清理市直党政机关、事业单位的资产，机关部门不再拥有资产经营权。用好省对县(市)激励型财政转移支付机制，合理调整市与区的财权关系，调动县（市、区)生财理财的积极性。

　　四、扩大对外开放和经济合作，提升西南出海通道战略地位

　　千方百计招商引资。发挥我市深水良港、丰富的海洋与农业资源和地处大西南出海通道的优势，努力实施外向带动战略，扩大利用外资规模，提高利用外资质量和水平。落实招商引资责任制，每个县(市、区)实际吸收外商直接投资争取超过1000万美元。落实各级鼓励外商投资和保护外商企业的各项政策，建立外商投资重点项目全程代办制和一般项目集中受理制。做到既要政府加强组织，又要体现以企业为主体，积极创新招商引资方式，提高招商引资工作实效。大力支持现有外资企业增资扩产。

　　加强区域经济合作。抓住“泛珠三角”区域合作、西部大开发和建立中国东盟自由贸易区带来的巨大商机，发挥区域枢纽作用，主动加强与珠江三角洲、大西南地区的对接与协作。积极用好内地与港、澳建立更紧密经贸关系安排的政策，加强与港澳地区的经济合作。积极开展与东盟地区的经济文化交流与合作。增进与国外友好城市交往，扩大湛江在海外知名度。

　　努力扩大外贸出口。主动适应国家出口退税政策调整，及时采取相应对策和措施，调整外贸出口结构。进一步改善口岸环境，推行电子监管和服务，完善“大通关”设施和机制，对依法经营、诚实守信的企业开辟进出口“绿色通道”。充分调动企业开发海外市场、扩大出口规模的积极性。落实鼓励和促进出口的各项政策，重点扶持出口1000万美元以上的企业。为更多符合条件的民营企业申报进出口经营权。扩大水产品、机电产品、纺织品、桉木片、富贵竹等大宗产品的出口市场份额，开拓花卉、蔬菜、水果等农产品出口市场。积极创造条件申报设立国家级出口加工区。

　　发展现代服务业和信息业。按照城市发展总体规划，实行新区开发和旧城改造并重，优化第三产业布局，拓展服务业发展空间，推进第三产业提档升级。发展连锁经营、物流配送、电子商务等现代流通与服务方式，发展壮大金融、证券、保险、咨询等服务业。搞好商业网点布局规划，培育大型中高级批发市场和大型购物中心。加强社区服务设施建设，发展各类便民、利民的社区服务项目。大力发展港口集装箱运输业和公路、铁路货运业，构建联系粤西、珠三角、大西南和东盟市场的石化、矿产、粮食和水产品等物流中心。发展信息产业，加强信息基础设施和信息化环境建设，推动信息技术在全市各行各业广泛应用，积极发展软件业和信息服务业。

　　发展壮大旅游业。广泛开展旅游优质服务年活动。以滨海风光、红土文化、热带亚热带风情、海鲜美食等特色资源为重点，加大旅游宣传推介力度，主动融入省港澳、粤桂琼大旅游区。加强旅游设施建设和旅游产品开发，完善主要风景区设施，抓好旅游资源的保护和开发。建立和完善城市旅游服务设施体系。建设旅游商品专卖店和美食城，发展旅游经济。继续举办大型醒狮比赛，扩大湛江民间艺术的影响。湖光岩风景区要强化保护，优化景观，积极申报世界地质公园。

　　五、加快基础设施建设，增强经济社会发展承载力

　　加快港口和交通道路建设。推进湛江港30万吨级航道、20万吨级铁矿石码头、流沙港码头等建设工程；认真做好东海岛深水岸线的利用规划，严格按照《港口法》加强港域管理。抓好渝湛高速公路湛江段、海湾大桥、疏港公路和国道207线良垌至遂溪段改扩建工程，新改建县通镇二级公路150公里。建设市、县(市、区)枢纽站场5个，新建市区公共汽车站场。力争海湾大桥二期工程列入今年省交通建设计划并动工建设。配合有关部门做好茂湛铁路、黎湛铁路复线河唇至湛江段和同三高速公路源水至徐闻段建设的前期工作。

　　加强城市规划和管理。积极开展创建国家卫生城市、国家园林城市和国家环保模范城市活动，营造优良的人居环境。实施湛江市城市近期建设规划和城市绿地系统规划；抓紧完成城市总体规划、城镇体系规划编制和报批，开展城市各专项规划和分区规划的编制以及城市控制性详细规划的修编工作。城市规划加强绿线管制。适时启动霞山洪屋路、赤坎南华市场旧城片区和“城中村”改造。强化规划对房地产的调控，引进新型住宅小区建设理念和模式，改善市民居住条件，拓展住房消费，促进房地产业健康发展。规范城市管理事权与责任，完善“统一领导、各司其职、规范管理、强化基层”的城市管理格局。清缴历年所欠的土地出让金和市政建设配套费。组建城市管理行政执法局，相对集中城市管理行政处罚权，加强城市有序管理。

　　推进市政基础设施建设。市政建设项目要积极有为，量力而行，加快速度，确保质量。今年市区市政建设项目共37项，投资10亿元。年内完成椹川大道、机场路、东新路和北桥公园、渔港公园、寸金路与铁路立交桥工程项目；加快南国热带花园、录塘河生态公园、赤坎水库公园、录塘路、百蓬路、金沙湾观海长廊二期、赤坎江、南柳河中游河道整治和冯村生活垃圾场二期填埋区建设；启动湛江大道建设和东坡荔园、滨湖公园改造前期工作；重新规划并整治霞湖公园及周边环境，营造市区绿心。继续依法拆除违章和影响市容市貌的建筑物，拆除市区旧殡仪馆，并做到拆除一片，绿化一片，提高城市绿化覆盖率、绿地率和人均公园绿地面积；年内建设绿地面积100万平方米，实现市区人均拥有公园绿地8平方米。新建一批垃圾转运站和公厕，更新改造一批环卫设施。各县(市)城区和镇要加强市政设施建设和市容环境卫生管理。

　　高度重视环保生态工作。围绕“以人为本、干净整洁、绿树成荫、生态平衡”的环保目标，增强市民的环保意识和自觉行为，开展群众性绿色单位创建活动。搞好城市环境综合整治，提高城市污水和垃圾集中处理率。改造城市水系，加快“三湖四河”环境治理。开工建设湛江电厂一期脱硫工程。对市区饮食业强制实施油改气计划。强化城市噪声、医疗垃圾和危险化学品的监督管理。完成赤坎污水处理厂建设并投入使用，动工建设霞山污水处理厂，各县（市）抓紧污水处理厂选址立项等前期工作。广泛推行清洁生产，加强工业污染治理，依法关闭污染严重企业。重视农业和农村污染的防治，大力推进生态示范区、生态示范村（镇、场、园）创建活动。实施污染物入海总量控制，整治东海岛沿岸的养殖污染，保护东海岛旅游区。开展创建林业生态县工作。进一步完善三岭山森林公园建设。加强矿产资源管理，公开拍卖矿产经营权。健全环境监测体系，加强地质环境保护和气象预测预报。

　　六、协调经济社会发展，推动文化大市建设

　　优先发展教育事业。加强基础教育，完善以县为主的农村义务教育管理体制，落实中小学教职工工资县长负责制。加大“防流控辍”和扶贫助学力度，巩固“普九”成果。按规划完成中小学布局调整，按时完成157所老区小学改造任务；继续推进市区学校改造工程，增加市区和县（市）城区学位，加快解决大班额问题。重视外来工子女入学。加快高中发展和示范性高中建设步伐，抓紧湛江一中扩建和市二中迁建，提高高中阶段教育入学率，增加优质学位。鼓励发展职业教育、民办教育，逐步形成公办和民办教育共同发展的新格局。积极支持在湛高校发展，扩大招生规模；组建高等职业技术学院。深化学校教学改革，推进中小学信息技术教育和英语教育。扩大推行中小学校长公开选拔聘用，抓好校长职级制和教职工全员聘用试点。

　　加快科技创新。继续开展党政领导班子和领导干部推动科技进步实绩考核。完善市级科技项目公开招标办法。建设好国家火炬计划湛江海洋产业基地，争取完成组建省级高新技术产业开发区工作。加快发展高新技术产业，鼓励企业建立各类技术开发机构，用高新技术和先进适用技术改造传统产业。继续建立农业科技示范基地。发挥科技中介服务机构的作用，培育技术市场。完善推进科技进步的人才、投入、成果转化评估机制和知识产权保护体系。发展科普教育，充实科普教育基地和科普长廊内涵。

　　繁荣文化事业。认真落实建设文化大市的总体部署，深化文化体制改革，加快文化事业和产业发展。进一步完善文化设施，加快广播电视中心建设，筹建市文化艺术中心，完善公用图书馆网络。大力发展广播电视事业，抓好广播电视网络的升级改造和联网，推进数字电视建设，加快自然村“村村通广播电视”步伐。实施文化精品工程，努力繁荣创作；弘扬传统文化艺术，打造一批地方文化品牌。鼓励和发展地方特有的雷剧，扶持粤剧南派艺术。抓实文化先进县创建工作，推进特色文化村建设，广泛开展群众性文化活动，大力开展文化下乡。积极运用市场机制优化配置文化资源，建立多元化的文化投融资机制，加快形成以文化重点产业为主导、相关产业联动发展的文化产业体系。支持图书业发展。推进经营性文化事业单位企业化改造，培育文化产业主体，使之逐步走向市场。积极引导文化消费，培育和规范文化市场。加强文物保护，发挥博物馆的社会功能。

　　发展公共卫生和体育事业。建立反应迅速、协调有力的突发公共卫生事件指挥体系和应急预警体系。推进疾病预防控制、卫生监督和医疗救治体系建设，重点抓好非典、禽流感等严重危害人民健康疾病的防控工作。积极开展创建国家卫生城市和卫生城镇（村）活动，加大力度治理垃圾、污水，从根本上改变脏、乱、差现象。加强农村卫生设施建设，改造20间乡镇薄弱卫生院。加大新型农村合作医疗试点工作力度，提高农村人口参加合作医疗覆盖率。完善妇幼保健体系，提高妇女儿童保健水平。振兴发展中医药事业。支持和引导民营医院的发展。加强食品、药品市场监管，保障人民群众食品卫生和用药安全。积极做好承办’2004世界第三区（亚澳）暨亚洲滑水锦标赛工作，认真办好第十届市运动会。广泛开展全民健身运动，争取新增1-2个县（市、区）达到省级体育先进县（市、区）标准。

　　加强人才工作。大力实施人才强市战略，大力培养、积极引进、合理使用各类人才。建设以中高级人才为重点的专业技术人才队伍，为经济建设和社会发展提供人才保障及智力支持。制定吸引留学生来湛创业的管理办法。把人才服务延伸到非公有制经济领域，努力开发农村人才资源，认真做好大中专毕业生就业工作。重视抓好人才培训和再教育工作。全面推进事业单位人事制度改革，推行聘用制，进行政府雇员制试点。

　　七、关注民生纾解民困，认真解决民众的实际困难

　　深入实施“十项民心工程”。政府组织，社会参与，突出重点，务求实效，认真实施全民安居、扩大与促进就业、农民减负增收、教育扶贫、济困助残、外来民工合法权益保护、全民安康、治污保洁、农村饮水、城乡防灾减灾等十项民心工程，切实改善人民群众生产生活条件。满腔热情为基层群众特别是困难群众排忧解难，确保全市城乡群众无饥寒。健全和完善社会保障体制。依法征缴社保费，清缴欠费，管好用好社会保障资金，实现社会保险市级统筹。重点抓好个体私营等非公有制企业和灵活就业人员的参保工作。完善失业保险制度，做好下岗职工基本生活保障与失业保险并轨的后续工作。完善医疗保险管理和配套政策措施，协调好医患保之间关系。完善最低工资制度、工资指导线制度、劳动力市场工资指导价位制度。

　　努力扩大和促进就业。坚持劳动者自主就业、市场调节就业、政府促进就业的方针，把改善创业环境和增加就业岗位作为政府工作的重要目标。认真落实社保补贴、就业服务等各项扶持政策，政府出资开发公益性岗位，对“4050”等就业困难人员实施援助，千方百计提高就业率。年内实现新增就业岗位3万个，下岗失业人员再就业2万人以上。加强就业和再就业培训，年内培训下岗失业人员1万人次。扩大市高级技工学校和新建市第二技工学校教学校区。完善公共就业服务体系，逐步建立统一的劳动力市场信息网络，重点抓好市区街道劳动保障事务所和社区工作站，开展社区就业和再就业服务。花更大力气组织农村劳动力外出务工，年内培训农村劳动力2万人，劳务输出17万人。

　　加强扶贫济困助残工作。坚持开发式扶贫，集中力量帮助贫困群众发展有特色、有市场的种养业项目。继续开展党政机关、事业单位定点扶贫，动员全社会力量扶贫济困助残。各级财政解决相应资金，开拓筹资渠道，重点解决困难群众、残疾人的低保救济和打官司难、住房难、看病难、子女入学难、社会保障难等“五难”问题。完善农村“五保户”生活保障制度，确保供养资金，用三年左右时间，全部建好村级敬老院。继续按计划兑现历年所欠农民甘蔗款。做好减免农村困难家庭子女义务教育阶段书杂费工作，提倡市直财政供养人员资助困难家庭子女入学。做好农村救灾救济工作，确保受灾群众生活有保障。维护残疾人权益，加快市残疾人康复中心建设。

　　稳步实施安居工程。切实搞好农村危房和茅草房改造，逐步改善农村困难户居住条件。落实各级有关政策，积极用好上级专项资金，改建水库移民危房5000户。妥善解决安置地点、建房用地、配套设施等问题，继续做好海上连家船渔民上岸安置工作。通过统筹建设廉租房、经济适用房和利用公房改建等途径，着力解决城镇特困户住房问题。有计划地进行市区旧城、危房改造，按政策做好拆迁户的安置补偿工作。

　　八、加强精神文明和民主法制建设，创造稳定和谐的社会环境

　　加强精神文明建设。积极开展社会公德、职业道德和家庭美德教育，弘扬爱国主义、集体主义、社会主义和艰苦创业精神，倡导尊老爱幼、助人敬贤、见义勇为的良好风尚，引导城乡居民形成科学、健康、文明的生活方式。开展“新时期湛江人精神”大讨论，大力培育和弘扬新时期湛江人精神。深入开展各项群众性精神文明创建活动，加强文明城市、文明村镇、文明社区、文明行业、文明单位建设，提高全社会的文明水平。全面启动创建全国文明城市活动，力争2005年实现目标。加强民主法制建设。自觉接受人大及其常委会的监督，认真执行人大及其常委会的决议、决定，支持人大代表依法行使职权，及时向人大及其常委会报告工作。加强与人民政协协商，接受政协的民主监督。认真办理人大代表议案、建议和政协委员提案，不断改进工作。主动听取民主党派、工商联、无党派人士的意见，发挥工会、共青团、妇联等群众团体的民主参与和民主监督作用。同时，要接受新闻舆论和社会公众监督。完善村民自治，加强社区民主法制建设，抓好基层“民主法治示范村”创建工作。坚持和完善以职代会为基本形式的企业民主管理制度，确保人民群众依法直接行使民主权利。加强法制宣传教育，做好行政复议、行政诉讼工作，提高政府和公民运用法律解决问题的能力，通过法定渠道加强对政府机关和工作人员的监督。

　　巩固计生和殡改成果。坚持党政一把手亲自抓、负总责及综合治理人口问题的原则，完善和落实人口与计生目标管理责任制，坚持“一票否决权”制度。开展“计生优惠政策落实年”活动。建立依法管理、村（居）民自治、优质服务、政策推动、综合治理的工作新机制。加强计划生育基层组织建设，推动工作重心下移。加快计生综合改革和信息化建设步伐，提高计生优质服务与管理水平。实行责任追究制度，切实解决漏报、瞒报、虚报问题。重视做好老龄工作。巩固殡改成果，完善市新殡仪馆设施，提高全市火化率。

　　强化社会治安工作。认真落实社会治安综合治理的各项措施，保障人民群众安居乐业。坚持“严打”方针不动摇，依法严厉打击各种刑事犯罪和黑恶势力，坚决扫除黄赌毒等社会丑恶现象，净化社会环境。加大打击“私彩”和禁毒收戒工作力度。强化基层基础工作，深入开展安全文明小区和无刑事案件社区创建活动，进一步发挥群防群治作用。加强劳动教养工作，重视劳教场所设施建设，做好刑释解教人员安置帮教工作，预防和减少重新违法犯罪。切实做好信访和人民调解工作，及时化解各种社会矛盾，依法妥善处置群体性、突发性事件，保持社会大局和谐稳定。

　　切实加强安全生产。全面贯彻《安全生产法》和国务院《关于进一步加强安全生产工作的决定》。进一步完善和落实安全生产各项政策措施，落实企业的安全生产主体责任，大力推进安全生产监管体制、安全生产法制和执法队伍“三项建设”，建立安全生产长效机制，加强对重点行业、地区和企业的安全生产监督管理，深入开展重大危险源的普查和监控，切实防止重特大安全事故发生，保障国家和群众生命财产安全。

　　整顿和规范市场经济秩序。坚决取消妨碍市场开放和公平竞争的行政审批事项，依法规范行政审批行为。加大市场监管力度，重点抓好食品、药品市场的专项整治，依法严厉打击制售假冒伪劣商品、走私贩私、偷税漏税、传销和变相传销等违法犯罪行为。整治价格垄断、价格欺诈等不正当竞争行为。积极探索建立城市交通和运输市场秩序长效管理机制，为乘客和货主提供安全、便捷的服务。严肃查处各种乱收费、乱罚款行为。公路上严禁违规设卡，力争年内实现公路无“三乱”。加强企业和公民的信用教育，完善适应市场经济的信用制度。

　　全面做好社会各项工作。继续抓好全民国防教育，增强全民国防意识，做好国防动员和民兵预备役、人民防空工作。深入开展双拥共建活动，做好优抚安置工作，帮助驻湛部队解决实际问题。完善统计制度，认真开展第一次经济普查。推行价格听证制度，健全价格监测预警机制。强化审计监督工作，加大对财政资金和国有资产的监管力度。出版《湛江市志》。加快建设数字化档案，开展政府文件公开阅览服务。进一步做好民族、宗教、对台、城调、企调等工作，促进社会各项事业协调发展。

　　九、坚持以民为本，建设“为民、务实、清廉”政府

　　全市各级政府和各级领导、全体公务员要强化以民为本、执政为民意识，坚持做到权为民所用，情为民所系，利为民所谋，一心对人民负责，一切为了人民的利益。

　　坚持依法行政。各级政府要严格按照法定权限和程序行使权力，履行职责，共同建设行为规范、运转协调、公正透明、廉洁高效的行政管理体制。坚持从严治政，奖惩分明，实行执法责任制、过错追究制和行政赔偿制，做到有权必有责，用权受监督，侵权要赔偿，违法受制裁，坚决纠正损害群众利益的不正之风。认真贯彻实施《行政许可法》，公布行政许可事项和实施主体，大力推动政府由传统管理型向现代服务型转变。规范各级各部门事权、财权、人事权和监督权。完善建设工程招标投标、经营性土地招标拍卖出让、国有产权交易、药品招标和政府采购等制度。

　　推进政府职能转变。认真履行经济调节、市场监管、社会管理和公共服务的政府职能。创新政府管理方式，全面推行政务公开，增强政府工作透明度，在市区增设大型政务公开栏，在市主要宣传媒体开辟政务公开和政务咨询栏。建立政府信息发布和政府发言人制度。完善重大问题和与老百姓利益密切相关事项的议事规则和决策程序，建立健全群众建议征集、重大事项社会公示和社会听证、专家咨询制度，提高决策的透明度和公众参与度，使政府的决策与工作更好地体现人民群众的意愿和利益。继续开展“振兴湛江——市民创意奖”活动。改进行政服务方式和手段，推行电子政务和“一站式”网上受理、审批及联合年审年检等，方便基层和群众办事。市政府行政服务中心要进一步规范办事程序，集中办事项目，抓好跟踪落实。

　　加强政府作风和廉政建设。各级领导干部要树立求真务实精神和正确的政绩观，力戒官僚主义、形式主义和奢侈浪费，把更多的时间精力、更多的财力物力用在推进经济建设、社会事业发展和解决民众实际问题上。心中要时时装着湛江百姓的福祉，深入基层体察民情，虚心倾听民众心声，善于采纳民众意见，坚决维护民众权利，亲力亲为解决民众困难。坚持抓好机关作风测评，在政府部门开展行风评议，进一步改进机关作风，增强服务意识，提高行政效率。倡导“干净干事”，加强公务员队伍建设，提高公务员素质。始终保持干事创业、甘于奉献的精神状态，切实把各项工作落到实处，抓出实效，干出水平。坚持标本兼治，惩防并举，从严治政，持之以恒地开展反腐倡廉工作，加大查处违法违纪案件力度，强化对权力实施有效的监督和制约，确保廉政建设和反腐败各项任务落实到实处。

　　各位代表!湛江正处在全面建设小康社会、努力赶上全省率先基本实现社会主义现代化步伐的重要时期，任务艰巨而光荣，责任具体而重大。让我们在中共湛江市委的坚强领导下，努力实践“三个代表”重要思想和科学发展观，与时俱进开拓创新，发愤图强团结实干，为建设现代化新兴港口工业城市和美丽的海滨城市而努力奋斗！
